# *N*,*N*′-[(2,3,5,6-Tetra­methyl-*p*-phenyl­ene)dimethyl­ene]bis­[2-chloro-*N*-(2-chloro­ethyl)ethanamine]

**DOI:** 10.1107/S1600536809026300

**Published:** 2009-07-15

**Authors:** Guang-Zhou Wang, Ya-Yun Zhuang, Cheng-He Zhou

**Affiliations:** a Laboratory of Bioorganic & Medicinal Chemistry, School of Chemistry and Chemical Engineering, Southwest University, Chongqing 400715, People’s Republic of China

## Abstract

The title mol­ecule, C_20_H_32_Cl_4_N_2_, lies on an inversion center. A weak intra­molecular C—H⋯N hydrogen bond may, in part, influence the conformation of the mol­ecule.

## Related literature

For a related crystal structure, see: Yin *et al.* (2006 ▶). For general background to the pharmacological activity of nitro­gen mustards, see: Rachid *et al.* (2007 ▶); Duan *et al.* (2008 ▶); Zhou *et al.* (2009 ▶); Zhuang *et al.* (2008 ▶).
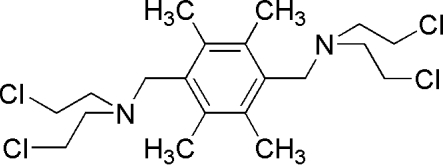


## Experimental

### 

#### Crystal data

C_20_H_32_Cl_4_N_2_
*M* *_r_* = 442.29
Monoclinic, 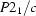

*a* = 13.6694 (14) Å
*b* = 9.751 (1) Å
*c* = 8.3997 (8) Å
β = 93.695 (2)°
*V* = 1117.27 (19) Å^3^
*Z* = 2
Mo *K*α radiation
μ = 0.54 mm^−1^
*T* = 298 K
0.16 × 0.12 × 0.10 mm

#### Data collection

Bruker SMART CCD diffractometer
Absorption correction: multi-scan (*SADABS*; Sheldrick,1996 ▶) *T*_min_ = 0.919, *T*_max_ = 0.948
13369 measured reflections
2732 independent reflections
2283 reflections with *I* > 2σ(*I*)
*R*_int_ = 0.031

#### Refinement

*R*[*F* ^2^ > 2σ(*F* ^2^)] = 0.048
*wR*(*F* ^2^) = 0.134
*S* = 1.04
2732 reflections
120 parameters
H-atom parameters constrained
Δρ_max_ = 0.46 e Å^−3^
Δρ_min_ = −0.25 e Å^−3^

### 

Data collection: *SMART* (Bruker, 2001 ▶); cell refinement: *SAINT-Plus* (Bruker, 2001 ▶); data reduction: *SAINT-Plus*; program(s) used to solve structure: *SHELXS97* (Sheldrick, 2008 ▶); program(s) used to refine structure: *SHELXL97* (Sheldrick, 2008 ▶); molecular graphics: *SHELXTL* (Sheldrick, 2008 ▶); software used to prepare material for publication: *SHELXTL*.

## Supplementary Material

Crystal structure: contains datablocks I, global. DOI: 10.1107/S1600536809026300/lh2858sup1.cif

Structure factors: contains datablocks I. DOI: 10.1107/S1600536809026300/lh2858Isup2.hkl

Additional supplementary materials:  crystallographic information; 3D view; checkCIF report

## Figures and Tables

**Table 1:** Hydrogen-bond geometry (Å, °)

*D*—H⋯*A*	*D*—H	H⋯*A*	*D*⋯*A*	*D*—H⋯*A*
C1—H1*C*⋯N1	0.96	2.43	3.159 (3)	133

**Fig. 1.**
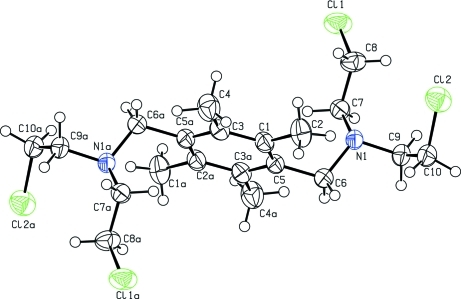
The molecular structure of (I), showing the atom-numbering scheme. Displacement ellipsoids are drawn at the 50% probability level [symmetry code: (a) -x, -y, -z+1].

## supplementary crystallographic information

### Comment

Nitrogen mustards such as chlorambucil and melphalan are cytotoxic chemotherapy agents, which are widely used in the treatment of a variety of malignant diseases. As bifunctional DNA-alkylating agents, nitrogen mustards are able to crosslink cellular DNA and thereby interfere with the DNA replication (Rachid *et al.*, 2007; Duan *et al.*, 2008; Zhuang *et al.*, 2008; Zhou *et al.*, 2009). The title compound (I), was obtained by the chlorination of the corresponding diol. Here we present the crystal structure of (I) (Fig. 1).

The title molecule lies on an inversion center. A weak intramolecular C—H···N hydrogen bond may, in part, influence the conformation of the molecule.

### Experimental

To a stirred solution of 1,4-bis(bromomethyl)-2,3,5,6-tetramethylbenzene (6.40g, 20mmol) in absolute alcohol (30mL) at 348K potassium carbonate (5.53g, 40mmol) and 2,2'-azanediyldiethanol (4.21g, 40mmol) were added. The progress of the reaction was monitored by TLC. The mixture was filtered to remove the inorganic salts, the solvent was concentrated under reduced pressure and recrystallization from absolute alcohol gave the intermediate2,2',2'',2'''-(2,3,5,6-tetramethyl-*p*-phenylene)bis (methylene) bis (azanetriyl)tetraethanol (Yield: 5.23g, 71.0%; white solid; Mp., 417-418K). Sulfonyl chloride (40mL) was added dropwise to the intermediate (3.68g, 10mmol) in  an ice-salt bath and then the mixture was stirred slowly at gentle reflux for three hours. Sulfonyl chloride was removed under reduced pressure, after cooling, water was added cautiously, and then the mixture was neutralized with NaHCO_3_. The suspension was filtered and washed with chloroform. The organic layer was washed with water, dried over anhydrous Na_2_SO_4_ and the solvent was removed in vacuo. The resulting residue was recrystallized from chloroform to give the title compound (Yield: 3.83g, 86.7%; white solid; Mp. 389-340K).

### Refinement

Hydrogen atoms were placed in calculated positions with C—H = 0.93Å (aromatic), 0.97Å (methylene) and 0.96Å (methyl) with *U*_iso_(H) = 1.2*U*_eq_(C) (aromatic and methylene C) or 1.5*U*_eq_(C) (methyl C).

### Crystal data

**Table:**

C_20_H_32_Cl_4_N_2_	*F*(000) = 468
*M**_r_* = 442.29	*D*_x_ = 1.315 Mg m^−^^3^
Monoclinic, *P*2_1_/*c*	Mo *K*α radiation, λ = 0.71073 Å
Hall symbol: -P 2ybc	Cell parameters from 4998 reflections
*a* = 13.6694 (14) Å	θ = 2.6–28.0°
*b* = 9.751 (1) Å	µ = 0.54 mm^−^^1^
*c* = 8.3997 (8) Å	*T* = 298 K
β = 93.695 (2)°	Block, white
*V* = 1117.27 (19) Å^3^	0.16 × 0.12 × 0.10 mm
*Z* = 2	

### Data collection

**Table:**

Bruker SMART CCD diffractometer	2732 independent reflections
Radiation source: fine-focus sealed tube	2283 reflections with *I* > 2σ(*I*)
graphite	*R*_int_ = 0.031
φ and ω scans	θ_max_ = 28.3°, θ_min_ = 2.6°
Absorption correction: multi-scan (*SADABS*; Sheldrick,1996)	*h* = −17→17
*T*_min_ = 0.919, *T*_max_ = 0.948	*k* = −12→12
13369 measured reflections	*l* = −11→11

### Refinement

**Table:**

Refinement on *F*^2^	Primary atom site location: structure-invariant direct methods
Least-squares matrix: full	Secondary atom site location: difference Fourier map
*R*[*F*^2^ > 2σ(*F*^2^)] = 0.048	Hydrogen site location: inferred from neighbouring sites
*wR*(*F*^2^) = 0.134	H-atom parameters constrained
*S* = 1.04	*w* = 1/[σ^2^(*F*_o_^2^) + (0.0809*P*)^2^ + 0.1826*P*] where *P* = (*F*_o_^2^ + 2*F*_c_^2^)/3
2732 reflections	(Δ/σ)_max_ = 0.001
120 parameters	Δρ_max_ = 0.46 e Å^−^^3^
0 restraints	Δρ_min_ = −0.25 e Å^−^^3^

### Special details

**Table:**

Geometry. All e.s.d.'s (except the e.s.d. in the dihedral angle between two l.s. planes) are estimated using the full covariance matrix. The cell e.s.d.'s are taken into account individually in the estimation of e.s.d.'s in distances, angles and torsion angles; correlations between e.s.d.'s in cell parameters are only used when they are defined by crystal symmetry. An approximate (isotropic) treatment of cell e.s.d.'s is used for estimating e.s.d.'s involving l.s. planes.
Refinement. Refinement of *F*^2^ against ALL reflections. The weighted *R*-factor *wR* and goodness of fit *S* are based on *F*^2^, conventional *R*-factors *R* are based on *F*, with *F* set to zero for negative *F*^2^. The threshold expression of *F*^2^ > σ(*F*^2^) is used only for calculating *R*-factors(gt) *etc*. and is not relevant to the choice of reflections for refinement. *R*-factors based on *F*^2^ are statistically about twice as large as those based on *F*, and *R*- factors based on ALL data will be even larger.

### Fractional atomic coordinates and isotropic or equivalent isotropic displacement parameters (Å^2^)

**Table:**

	*x*	*y*	*z*	*U*_iso_*/*U*_eq_	
C1	0.12764 (15)	0.2248 (2)	0.6112 (3)	0.0550 (5)	
H1A	0.1026	0.2650	0.7046	0.083*	
H1B	0.1296	0.2930	0.5290	0.083*	
H1C	0.1926	0.1906	0.6366	0.083*	
C2	0.06149 (12)	0.10771 (19)	0.5531 (2)	0.0396 (4)	
C3	−0.03006 (12)	0.13651 (18)	0.4753 (2)	0.0400 (4)	
C4	−0.06146 (16)	0.2853 (2)	0.4533 (3)	0.0578 (5)	
H4A	−0.0419	0.3364	0.5477	0.087*	
H4B	−0.1314	0.2897	0.4345	0.087*	
H4C	−0.0309	0.3237	0.3637	0.087*	
C5	0.09103 (12)	−0.02818 (19)	0.57881 (19)	0.0380 (4)	
C6	0.18937 (12)	−0.0575 (2)	0.6671 (2)	0.0426 (4)	
H6A	0.1939	−0.0056	0.7658	0.051*	
H6B	0.1927	−0.1541	0.6944	0.051*	
C7	0.27229 (13)	−0.08753 (18)	0.4172 (2)	0.0381 (4)	
H7A	0.2054	−0.1096	0.3803	0.046*	
H7B	0.3099	−0.1720	0.4238	0.046*	
C8	0.31611 (16)	0.0100 (2)	0.3029 (2)	0.0476 (4)	
H8A	0.3821	0.0342	0.3425	0.057*	
H8B	0.2774	0.0934	0.2952	0.057*	
C9	0.36606 (12)	−0.04271 (19)	0.6687 (2)	0.0403 (4)	
H9A	0.4158	−0.0715	0.5986	0.048*	
H9B	0.3577	−0.1159	0.7448	0.048*	
C10	0.40134 (13)	0.0836 (2)	0.7575 (2)	0.0454 (4)	
H10A	0.4551	0.0592	0.8332	0.055*	
H10B	0.3487	0.1199	0.8171	0.055*	
N1	0.27367 (9)	−0.02246 (15)	0.57401 (16)	0.0353 (3)	
Cl1	0.31962 (4)	−0.06609 (6)	0.10972 (6)	0.0624 (2)	
Cl2	0.44162 (4)	0.21356 (5)	0.62479 (7)	0.05813 (19)	

### Atomic displacement parameters (Å^2^)

**Table:**

	*U*^11^	*U*^22^	*U*^33^	*U*^12^	*U*^13^	*U*^23^
C1	0.0412 (10)	0.0543 (12)	0.0691 (14)	−0.0048 (8)	0.0002 (9)	−0.0143 (10)
C2	0.0307 (8)	0.0500 (10)	0.0387 (9)	−0.0023 (7)	0.0059 (6)	−0.0025 (7)
C3	0.0325 (8)	0.0471 (10)	0.0412 (9)	0.0009 (7)	0.0081 (7)	0.0014 (7)
C4	0.0443 (11)	0.0506 (11)	0.0779 (15)	0.0040 (8)	0.0004 (10)	0.0066 (10)
C5	0.0279 (8)	0.0535 (10)	0.0329 (8)	0.0005 (7)	0.0044 (6)	0.0014 (7)
C6	0.0319 (8)	0.0623 (11)	0.0336 (8)	−0.0002 (7)	0.0028 (6)	0.0061 (8)
C7	0.0364 (8)	0.0440 (9)	0.0341 (8)	−0.0054 (7)	0.0033 (6)	−0.0018 (7)
C8	0.0630 (12)	0.0471 (10)	0.0329 (9)	−0.0063 (9)	0.0049 (8)	−0.0025 (8)
C9	0.0309 (8)	0.0485 (10)	0.0410 (9)	0.0037 (7)	−0.0019 (7)	0.0027 (7)
C10	0.0373 (9)	0.0608 (12)	0.0376 (9)	−0.0038 (8)	−0.0024 (7)	−0.0015 (8)
N1	0.0272 (6)	0.0470 (8)	0.0316 (7)	−0.0005 (5)	0.0007 (5)	−0.0014 (6)
Cl1	0.0788 (4)	0.0759 (4)	0.0331 (3)	−0.0251 (3)	0.0084 (2)	−0.0071 (2)
Cl2	0.0622 (3)	0.0514 (3)	0.0611 (3)	−0.0097 (2)	0.0064 (2)	−0.0031 (2)

### Geometric parameters (Å, °)

**Table:**

C1—C2	1.518 (3)	C6—H6B	0.9700
C1—H1A	0.9600	C7—N1	1.461 (2)
C1—H1B	0.9600	C7—C8	1.504 (2)
C1—H1C	0.9600	C7—H7A	0.9700
C2—C5	1.398 (3)	C7—H7B	0.9700
C2—C3	1.402 (2)	C8—Cl1	1.7874 (19)
C3—C5^i^	1.403 (2)	C8—H8A	0.9700
C3—C4	1.521 (3)	C8—H8B	0.9700
C4—H4A	0.9600	C9—N1	1.462 (2)
C4—H4B	0.9600	C9—C10	1.503 (3)
C4—H4C	0.9600	C9—H9A	0.9700
C5—C3^i^	1.403 (2)	C9—H9B	0.9700
C5—C6	1.520 (2)	C10—Cl2	1.798 (2)
C6—N1	1.473 (2)	C10—H10A	0.9700
C6—H6A	0.9700	C10—H10B	0.9700
			
C2—C1—H1A	109.5	N1—C7—C8	108.58 (14)
C2—C1—H1B	109.5	N1—C7—H7A	110.0
H1A—C1—H1B	109.5	C8—C7—H7A	110.0
C2—C1—H1C	109.5	N1—C7—H7B	110.0
H1A—C1—H1C	109.5	C8—C7—H7B	110.0
H1B—C1—H1C	109.5	H7A—C7—H7B	108.4
C5—C2—C3	120.14 (16)	C7—C8—Cl1	110.64 (13)
C5—C2—C1	120.23 (16)	C7—C8—H8A	109.5
C3—C2—C1	119.63 (17)	Cl1—C8—H8A	109.5
C2—C3—C5^i^	119.59 (16)	C7—C8—H8B	109.5
C2—C3—C4	118.96 (16)	Cl1—C8—H8B	109.5
C5^i^—C3—C4	121.45 (16)	H8A—C8—H8B	108.1
C3—C4—H4A	109.5	N1—C9—C10	113.44 (14)
C3—C4—H4B	109.5	N1—C9—H9A	108.9
H4A—C4—H4B	109.5	C10—C9—H9A	108.9
C3—C4—H4C	109.5	N1—C9—H9B	108.9
H4A—C4—H4C	109.5	C10—C9—H9B	108.9
H4B—C4—H4C	109.5	H9A—C9—H9B	107.7
C2—C5—C3^i^	120.26 (15)	C9—C10—Cl2	111.79 (13)
C2—C5—C6	119.41 (16)	C9—C10—H10A	109.3
C3^i^—C5—C6	120.33 (16)	Cl2—C10—H10A	109.3
N1—C6—C5	113.28 (14)	C9—C10—H10B	109.3
N1—C6—H6A	108.9	Cl2—C10—H10B	109.3
C5—C6—H6A	108.9	H10A—C10—H10B	107.9
N1—C6—H6B	108.9	C7—N1—C9	113.11 (13)
C5—C6—H6B	108.9	C7—N1—C6	114.36 (13)
H6A—C6—H6B	107.7	C9—N1—C6	110.96 (13)
			
C5—C2—C3—C5^i^	−1.1 (3)	C3^i^—C5—C6—N1	110.03 (18)
C1—C2—C3—C5^i^	179.79 (17)	N1—C7—C8—Cl1	178.35 (12)
C5—C2—C3—C4	178.23 (17)	N1—C9—C10—Cl2	−69.96 (18)
C1—C2—C3—C4	−0.9 (3)	C8—C7—N1—C9	−86.20 (18)
C3—C2—C5—C3^i^	1.1 (3)	C8—C7—N1—C6	145.47 (16)
C1—C2—C5—C3^i^	−179.79 (17)	C10—C9—N1—C7	138.52 (16)
C3—C2—C5—C6	−178.45 (15)	C10—C9—N1—C6	−91.42 (19)
C1—C2—C5—C6	0.7 (3)	C5—C6—N1—C7	−54.9 (2)
C2—C5—C6—N1	−70.5 (2)	C5—C6—N1—C9	175.66 (15)

Symmetry codes: (i) −*x*, −*y*, −*z*+1.

### Hydrogen-bond geometry (Å, °)

**Table:**

*D*—H···*A*	*D*—H	H···*A*	*D*···*A*	*D*—H···*A*
C1—H1C···N1	0.96	2.43	3.159 (3)	133
